# Ultrasensitive CRISPR/Cas12a-Based System for Detection of *Bla*_OXA-1_ Gene in Antibiotic-Resistant Microorganisms

**DOI:** 10.3390/cimb47040238

**Published:** 2025-03-29

**Authors:** Marina Tyumentseva, Aleksandr Tyumentsev, Anna Prelovskaya, Andrey Akinin, Yulia Mikhailova, Andrey Shelenkov, Anna Panevina, Vasiliy Akimkin

**Affiliations:** Central Research Institute of Epidemiology, Novogireevskaya Str., 3a, 111123 Moscow, Russia; tymencev@cmd.su (A.T.); prelovskaya@cmd.su (A.P.); akinin@cmd.su (A.A.); mihailova@cmd.su (Y.M.); Panevina@cmd.su (A.P.); vgakimkin@yandex.ru (V.A.)

**Keywords:** antibiotic-resistant microorganisms, ESBL, *bla*_OXA-1_, PCR, CRISPR/Cas12a, detection

## Abstract

The *bla*_OXA-1_ gene encodes an oxacillin-hydrolyzing beta-lactamase of extended-spectrum beta-lactamase (ESBL)-producing microorganisms. The *bla*_OXA-1_ gene is found in the resistomes of some *Enterobacteriaceae*, *Morganellaceae*, *Pasteurellaceae*, *Moraxellaceae*, *Aeromonadaceae*, *Pseudomonadaceae*, *Yersiniaceae*, and *Vibrionaceae*. Most ESBL detection methods, including those to detect OXA-1-producing microorganisms, are time-consuming, and require specialized equipment and qualified personnel. Here, we report a new CRISPR(Clustered Regularly Interspaced Short Palindromic Repeats)/Cas12a-based detection assay coupled with polymerase chain reaction (PCR) to sensitively detect OXA-1-bearing microorganisms. The PCR-coupled CRISPR/Cas12a-based fluorescence assay includes (i) a pre-amplification step and (ii) a nucleic acid detection step. The pre-amplification step is based on a commonly used PCR, and the detection step is based on the CRISPR/Cas12a property to nonspecifically hydrolyze single-stranded DNA fluorescent reporter molecules. The pre-amplification step takes 65 min, and the detection step is shortened and takes only 5 min. The developed assay can easily detect single (1.25) copies of the *bla*_OXA-1_ gene in a reaction and is efficient not only in the detection of a *bla*_OXA-1_ model matrix but also in the detection of *bla*_OXA-1_-positive microorganisms. We hope that our assay has the potential to improve the monitoring of OXA-1-producing microorganisms and therefore contribute to mitigating the deadly global threat of antibiotic-resistant microorganisms.

## 1. Introduction

The rapid emergence of antibiotic-resistant microorganisms poses a significant threat to global public health, necessitating the development of innovative detection strategies [1]. Conventional methods, such as culture-based techniques, often lack the speed and sensitivity required for early identification, leading to delays in appropriate treatment [2]. Advanced molecular approaches, including next-generation sequencing (NGS) and CRISPR-based diagnostics, offer promising alternatives by enabling rapid and precise detection of resistance genes [3]. Furthermore, machine learning algorithms can enhance pathogen surveillance by predicting resistance patterns from genomic data [4]. The integration of these novel strategies into clinical practice is critical for combating the escalating antimicrobial resistance (AMR) crisis [5].

Extended-spectrum β-lactamases (ESBLs), particularly those conferring resistance to penicillins, cephalosporins, and aztreonam, represent a critical therapeutic challenge. The global prevalence of ESBL-producing *Enterobacteriaceae* [6] and their association with poor clinical outcomes [7] underscore the urgency for improved detection methods.

The *bla*_OXA-1_ gene encodes the oxacillin-hydrolyzing class D β-lactamase OXA-1. Originally identified as part of the RGN238 plasmid-borne transposon [8], *bla*_OXA-1_ now demonstrates widespread distribution across clinically relevant pathogens including *Acinetobacter baumannii*, *Escherichia coli*, *Klebsiella pneumoniae*, and *Vibrio cholerae* (https://card.mcmaster.ca/ontology/37796, accessed on 28 January 2025).

Current ESBL detection kits predominantly utilize agar diffusion and isoelectric focusing techniques [9,10]. However, the increasing diversity of ESBL variants (*bla*_SHV_, *bla*_TEM_, *bla*_CTX-M_) has diminished the reliability of phenotypic characterization [11], while these methods remain labor-intensive and equipment-dependent.

Consequently, there is an urgent need for novel, high-efficacy methods to detect antibiotic resistance genes—including *bla*_OXA-1_—in bacterial pathogens, leveraging advanced genetic technologies such as CRISPR/Cas systems.

CRISPR/Cas is a versatile genome-editing tool with broad applications, including functional genomics, biotechnology, gene therapy, and diagnostics. Studies have demonstrated its utility in precise pathogen detection and genotyping [12]. In 2018, it was discovered that the CRISPR-associated enzyme Cas12, upon binding to its target DNA, exhibits nonspecific single-stranded DNA cleavage. This property enabled the development of DETECTR (DNA Endonuclease Targeted CRISPR Trans Reporter), a nucleic acid detection platform for identifying viral or bacterial DNA. The system consists of Cas12a, a guide RNA, and a fluorescent reporter, requiring target pre-amplification (e.g., via PCR, Loop-Mediated Isothermal Amplification (LAMP), or Recombinase Polymerase Amplification (RPA)). DETECTR was first applied to detect human papillomavirus (HPV) [13]. Another CRISPR-based platform, SHERLOCK (Specific High-sensitivity Enzymatic Reporter unLOCKing), employs Cas13a for RNA detection. SHERLOCK can distinguish closely related viral strains (e.g., Zika and Dengue), genotype bacteria (*E. coli*, *P. aeruginosa*), and identify antibiotic resistance genes (e.g., in *K. pneumoniae*) with high specificity [14,15].

We present a novel CRISPR/Cas12a-based detection assay integrated with PCR for sensitive identification of OXA-1-producing microorganisms. The assay comprises two key steps: (i) target pre-amplification using conventional PCR and (ii) nucleic acid detection leveraging CRISPR/Cas12a’s collateral cleavage activity against single-stranded DNA fluorescent reporters. The optimized protocol achieves rapid detection, with pre-amplification completed in 65 min followed by a streamlined 5 min Cas12a-mediated detection phase. Demonstrating exceptional sensitivity, the assay reliably detects down to 1.25 copies of the *bla*_OXA-1_ gene per reaction and shows robust performance with both synthetic targets and clinical isolates containing *bla*_OXA-1_.

Traditional methods for detecting *bla*_OXA-1_ (real-time PCR, sequencing, microbiological assays) require long processing times (hours to days), specialized equipment, and skilled personnel. These limitations restrict their use in resource-limited settings and hinder rapid monitoring. With its relative simplicity and cost-effectiveness, this method can be deployed in clinical laboratories (for rapid infection diagnosis), hospital/agricultural settings (to track ESBL spread via food chains), and resource-limited regions. Early detection of OXA-1-producing pathogens will enable optimized antibiotic prescriptions (avoiding ineffective drugs), timely isolation of hospital carriers to prevent outbreaks, and improved global monitoring of resistant strains. The study addresses an urgent need in diagnosing resistant infections by providing a fast, accurate, and accessible method for *bla*_OXA-1_ detection. This is a crucial step in combating one of the greatest threats to modern medicine—antimicrobial resistance.

## 2. Materials and Methods

### 2.1. Oligonucleotides, Recombinant Proteins, and Buffers

All oligonucleotides, including ssDNA reporter molecules for the CRISPR/Cas12 fluorescence assay, primer sets, and guide RNAs (crRNAs), were purchased from GenTerra (Moscow, Russia). All nucleotide sequences used are listed in Table 1. Cas12a (LbCpf1) and Taq DNA polymerase were produced at the Central Research Institute of Epidemiology (Moscow, Russia).

The 10× Cas12 reaction buffer (100 mM Tris-HCl, 1 M NaCl, 50 mM MgCl_2_, pH 8.0 at 25 °C) and modified 10× HOLMES (one-hour low-cost multipurpose highly efficient system) Buffer 1 (400 mM Tris-HCl, 60 mM MgCl_2_, 400 mM glycine, 20 mM spermidine, 10 mM DTT, 0.01% Triton^®^ X-100, 4% PEG-8000, pH 8.5 at 25 °C) [16] were prepared using reagents purchased from Merck (Darmstadt, Germany) and Sisco Research Laboratories Pvt. Ltd. (Maharashtra, India).

### 2.2. Selection of Target Sequences in the Bla_OXA-1_ Antibiotic Resistance Gene to Develop Guide RNAs

To select target sequences in the *bla*_OXA-1_ antibiotic resistance gene and to develop guide RNAs, nucleotide sequences were aligned in the MEGA7.0.26 software [19] using the ClustalW «Align DNA» algorithm, as described elsewhere [20].

The guide RNAs were selected using Benchling (https://www.benchling.com/molecular-biology/, accessed on 31 January 2024). Briefly, the target *bla*_OXA-1_ gene fragment sequence (a 321 bp region of the *bla*_OXA-1_ gene, from 103 to 423 bp) was pasted into the «Design CRISPR guides: Import sequence» form, where «Raw Bases» were selected in the «Import from» field. In the «Design CRISPR guides: Guide parameters» form, «TTTN (AsCpf1/LbCpf1 5′ side)» was selected in the «PAM» (Protospacer Adjacent Motif) field, while other parameters were set as default.

### 2.3. Construction of a Model DNA Matrix Containing the Target bla_OXA-1_ Gene Fragment

The target *bla*_OXA-1_ gene fragment (a 321 bp region of the *bla*_OXA-1_ gene from 103 to 423 bp) was amplified using TaqF DNA polymerase (Central Research Institute of Epidemiology, Moscow, Russia) and oligonucleotides OXA-1_for_45 and OXA-1_rev_48 (GenTerra, Moscow, Russia) listed in Table 1. PCR was performed via (1) pre-denaturation at 95 °C for 15 min; (2) 35 cycles each consisting of denaturation at 95 °C for 15 s, annealing at 55 °C for 30 s, and elongation at 72 °C for 30 s; and (3) a final elongation at 72 °C for 5 min. The PCR products were then purified using the QIAquick PCR Purification Kit (QIAGEN, Hilden, Germany) according to the manufacturer’s protocol. The purified *bla*_OXA-1_ gene fragment was cloned into the pGEM^®^-T vector (Promega Corporation, Madison, WI, USA) according to the manufacturer’s protocol. Single clones containing the pGEM^®^-T plasmid with the target insert were obtained by chemical transformation of *E. coli* MACH1-T1 cells (Thermo Fisher Scientific, Waltham, MA, USA). Clones containing an insert of the required length were identified using PCR with M13 forward and M13 reverse primers (GenTerra, Moscow, Russia). Plasmid DNA was isolated using the QIAprep Spin Miniprep Kit (QIAGEN, Hilden, Germany) according to the manufacturer’s protocol, and the correctness of the inserted DNA was verified by capillary sequencing using the Applied Biosystems 3500xL Genetic Analyzer (Applied Biosystems, Waltham, MA, USA).

A bacterial clone containing the pGEM^®^-T-*bla*_OXA-1_ plasmid was grown overnight in Luria-Bertani broth (VWR, West Chester, PA, USA) containing 100 µg/mL ampicillin (AppliChem, Darmstadt, Germany), and the plasmid DNA was extracted using the QIAGEN Plasmid Midi Kit (QIAGEN, Hilden, Germany) according to the manufacturer’s protocol. The concentration of the pGEM^®^-T-*bla*_OXA-1_ plasmid was measured using the DeNovix dsDNA Ultra High Sensitivity Evaluation Kit (DeNovix Inc., Wilmington, DE, USA) according to the manufacturer’s protocol. The pGEM^®^-T-*bla*_OXA-1_ model matrix with concentrations ranging from 1.25 × 10^0^ copies/µL to 1.25 × 10^6^ copies/µL was prepared via serial dilutions.

### 2.4. Bla_OXA-1_ Target Gene Fragment Pre-Amplification

The target *bla*_OXA-1_ gene fragment pre-amplification was conducted using TaqF DNA polymerase (Central Research Institute of Epidemiology, Moscow, Russia) and oligonucleotides OXA-1_for_2 and OXA-1_rev_1 (GenTerra, Moscow, Russia) listed in Table 1. PCR was performed on a 30 µL mixture containing PCR master-mix, 10 pmol of each primer, and 1 µL of DNA template (either the pGEM^®^-T-*bla*_OXA-1_ model matrix with concentrations ranging from 1.25 × 10^0^ copies/µL to 1.25 × 10^6^ copies/µL or DNA isolated from clinical samples). The test tube without the *bla*_OXA-1_ gene (where 1 µL of nuclease-free water was added instead of the DNA template) served as the negative control for the pre-amplification step. PCR was performed via (1) pre-denaturation at 95 °C for 15 min; (2) 40 cycles each consisting of denaturation at 95 °C for 15 s, annealing at 55 °C for 30 s, and elongation at 72 °C for 30 s; and (3) a final elongation at 72 °C for 5 min. The size of the amplified *bla*_OXA-1_ gene fragment was 262 bp. To assess the effectiveness of the pre-amplification, the obtained fragment was visualized using agarose gel electrophoresis.

### 2.5. Bla_OXA-1_-Specific RNP Assembly and CRISPR/Cas12a Fluorescence Assay

The *bla*_OXA-1_-specific ribonucleoprotein complexes (RNPs) were assembled from 300 ng of the recombinant CRISPR/Cas12a LbCpf1 protein from *Lachnospiraceae bacterium* and 2 pmol of the guide RNA oligonucleotides (crRNAs) listed in Table 1 according to the standard protocol with slight modifications [21]. The LbCpf1 and crRNAs were mixed well and incubated at room temperature for 10 min to allow complex formation. The RNP complex obtained in this way was ready for detection of the *bla*_OXA-1_ antibiotic resistance gene.

To detect the antibiotic resistance gene *bla*_OXA-1_ using CRISPR/Cas12a RNPs, a reaction mixture must be prepared containing the following components: (i) 10× reaction buffer; (ii) the RNP; (iii) the fluorescent reporter molecule; (iv) the target DNA (the pre-amplified fragment of the *bla*_OXA-1_ antibiotic resistance gene); and (v) deionized mQ water. All DNA samples (either the pGEM^®^-T-*bla*_OXA-1_ model matrix with concentrations ranging from 1.25 × 10^0^ copies/µL to 1.25 × 10^6^ copies/µL or DNA isolated from clinical samples) were pre-amplified using PCR under the same conditions described in Section 2.4. Each Cas12a reaction mixture contained 300 ng of the Cas12a LbCpf1, 2 pmol of the crRNA, 10 pmol of the fluorescent reporter molecule, 5 µL of the PCR product, 2.5 µL of the 10× reaction buffer, and deionized mQ water to adjust the reaction volume to 25 µL. The negative control for the detection step consisted of a test tube containing 5 µL of the pre-amplification negative control instead of 5 µL of the PCR product.

The reaction mixtures containing all the necessary components were placed in a QuantStudio 5 thermal cycler (Thermo Fisher Scientific, Waltham, MA, USA), and the following reaction parameters were set: 60 cycles each consisting of an incubation step at 37 °C for 35 s and a fluorescence measurement step at 37 °C for 25 s. All experiments were performed in triplicate.

The signal-to-noise (S/N) ratio was calculated as the quotient of the fluorescence intensity from samples containing the pre-amplified target DNA to that of the negative control.

### 2.6. Detection of the Target Bla_OXA-1_ Gene in Real Samples

A total of 50 *bla*_OXA-1_-positive genomic DNA samples isolated from *E. cloacae*, *E. coli*, *K. pneumoniae*, *M. morganii*, *P. mirabilis*, *S. enterica*, and *S. marcescens* and 11 *bla*_OXA-1_-negative genomic DNA samples isolated from *A. baumannii*, *E. faecalis*, *E. faecium*, *P. aeruginosa*, *S. enterica*, and *S. aureus* were used to evaluate the developed assay performance. The presence/absence of the *bla*_OXA-1_ gene in the selected DNA samples was confirmed earlier by whole genome sequencing (Table S1). All samples originated from the same geographic region. Due to *bla*_OXA-1_’s high sequence identity across species and regions, an epidemiological assessment of the developed assay was beyond the scope of this study.

### 2.7. Data Processing

A two-way ANOVA (Analysis of Variance) with multiple comparisons was used to find significant differences between the fluorescent signals generated from the hydrolysis of the fluorescent reporter molecules and the mean S/N ratios obtained from different reaction conditions.

The graphs were generated using GraphPad Prism version 9.5.1.

## 3. Results

### 3.1. Design of CRISPR/Cas12a Fluorescence Assays and Guide RNA Selection

To select target sequences in the *bla*_OXA-1_ antibiotic resistance gene for guide RNA (gRNA) design, we aligned 101 nucleotide sequences of the *bla*_OXA-1_ gene from *C. cronae*, *C. freundii*, *C. meridianamericanus*, *C. portucalensis*, *C. youngae*, *E. bugandensis*, *E. hormaechei*, *E. coli*, *K. michiganensis*, *K. oxytoca*, *K. pneumoniae*, *K. quasipneumoniae*, *K. variicola*, *M. morganii*, *P. mirabilis*, *P. terrae*, *P. aeruginosa*, *R. planticola*, *S. enterica*, and *S. flexneri*. The analysis revealed the *bla*_OXA-1_ gene to be highly conserved, with all nucleotide sequences showing 100% identity (Table S2). For pre-amplification oligonucleotide design and guide RNA selection, we targeted a 321 bp region of the *bla*_OXA-1_ gene (positions 103 to 423).

For guide RNA target selection, we used Benchling (https://www.benchling.com/molecular-biology/, accessed 31 January 2024) to analyze the *bla*_OXA-1_ antibiotic resistance gene. We compiled a list of target regions with calculated specificity scores (Table S3) and selected three sequences: one with the highest score in the 1–100 nucleotide range, another in the 101–200 nucleotide range, and a third in the 201–321 nucleotide range.

### 3.2. Performance Analysis of the CRISPR/Cas12a Fluorescence Assay

Previous studies have established that guide RNAs exhibit variable efficiencies in CRISPR/Cas12a-based detection systems [22,23,24,25]. Based on these findings, we selected three specific guide RNAs (crRNA_*bla*_OXA-1__63, crRNA_*bla*_OXA-1__185, and crRNA_*bla*_OXA-1__221) for development of a detection system targeting the *bla*_OXA-1_ antibiotic resistance gene. The complete sequences of these guide RNAs are provided in Table 1.

**Figure 1:**
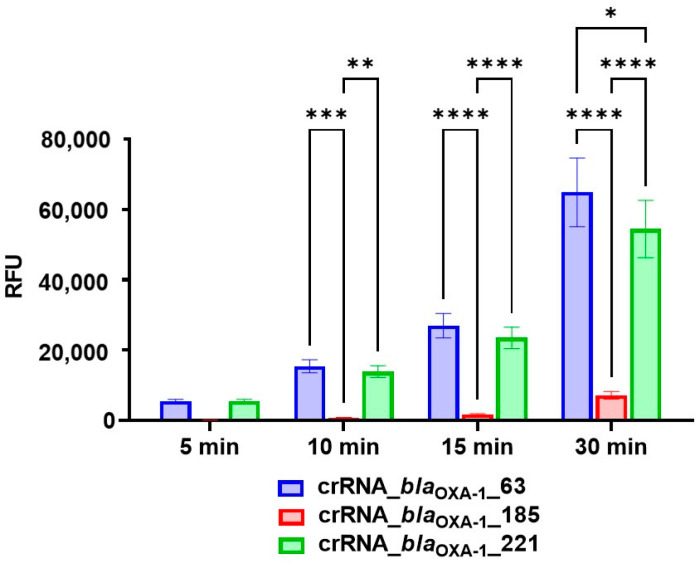
Fluorescence signal generated in the developed CRISPR/Cas12a assay for detection of the target gene using different guide RNAs. A 1.25 × 10^0^ copy number of the *bla*_OXA-1_-encoding model matrix was added per pre-amplification step in all conditions. Error bars represent the standard error of the mean (n = 3). RFU = relative fluorescence units. Asterisks denote *p*-values (* *p* ≤ 0.05, ** *p* ≤ 0.01, *** *p* ≤ 0.001, **** *p* ≤ 0.0001).

Our experimental results demonstrated that ribonucleoprotein (RNP) complexes incorporating all three selected guide RNAs could successfully detect single-copy (1.25 × 10^0^) targets of the *bla*_OXA-1_ gene following pre-amplification, albeit with varying efficiencies (Figure 1). Quantitative analysis revealed significantly stronger fluorescent signals (*p* = 0.0004) from reactions containing crRNA_*bla*_OXA-1__63 and crRNA_*bla*_OXA-1__221 compared with crRNA_*bla*_OXA-1__185 as early as the 10 min time point. This performance differential persisted through subsequent measurements, with crRNA_*bla*_OXA-1__185 demonstrating consistently lower signal intensity (*p* = 0.0014 vs. crRNA_*bla*_OXA-1__221). Notably, at the 30 min interval, reactions incorporating crRNA_*bla*_OXA-1__63 maintained a significant advantage over those with crRNA_*bla*_OXA-1__221 (*p* = 0.0106). Comprehensive statistical analysis of fluorescence signals generated through reporter molecule hydrolysis, including all significant inter-group differences, is presented in Table S4 and illustrated in Figure 1.

The target S/N ratio of 5 was achieved within 5–7 min when using RNPs containing either crRNA_*bla*_OXA-1__63 or crRNA_*bla*_OXA-1__221. In contrast, RNPs incorporating crRNA_*bla*_OXA-1__185 required 23 min to reach equivalent detection sensitivity.

**Figure 2:**
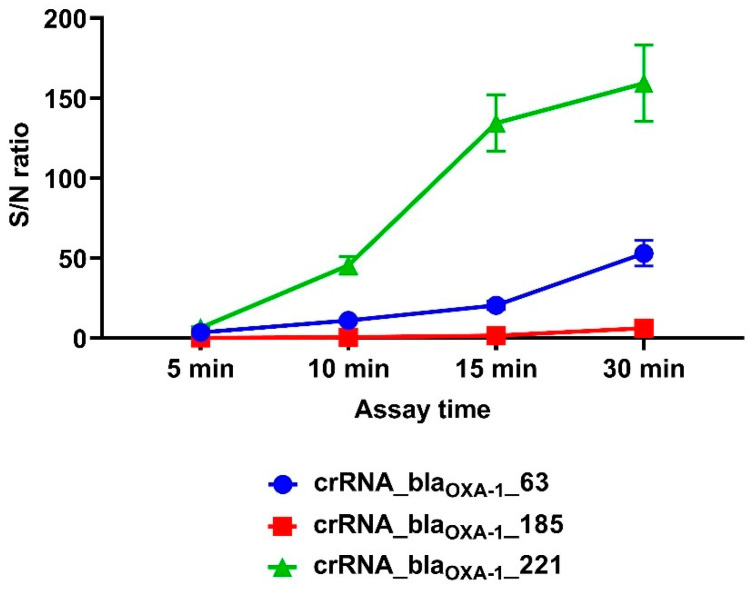
Signal-to-noise ratio of the developed CRISPR/Cas12a system for detection of the target gene using different guide RNAs. A 1.25 × 10^0^ copy number of the *bla*_OXA-1_-encoding model matrix was added per pre-amplification step in all conditions. Error bars represent the standard error of the mean (n = 3).

Among the three guide RNAs evaluated, crRNA_*bla*_OXA-1__221 demonstrated superior early assay performance, achieving a significantly higher S/N ratio of 45.48 by the 10 min time point compared with both crRNA_*bla*_OXA-1__63 (*p* = 0.0003) and crRNA_*bla*_OXA-1__185 (*p* < 0.0001). Notably, crRNA_*bla*_OXA-1__63 maintained a significant advantage over crRNA_*bla*_OXA-1__185 at the 15 min interval (S/N ratios of 20.60 vs. 1.65, respectively; *p* = 0.0429) (Figure 2). Complete statistical comparisons of S/N ratios across experimental conditions are provided in Table S5.

### 3.3. Assay Optimization

The sensitivity of CRISPR/Cas12a-based detection systems can be enhanced through multiple approaches, including the use of optimized fluorescent reporter molecules and assay buffers [13,16,17,18]. To investigate the impact of reporter molecule selection on assay performance, we systematically evaluated four distinct reporter systems: (i) the conventional ssDNA-FQ reporter [13], (ii) the TTATT-5C reporter [17], (iii) the 8C FQ-reporter [16], and (iv) the Stem-loop #10T reporter [18]. Complete sequences for all reporter molecules are provided in Table 1.

**Figure 3:**
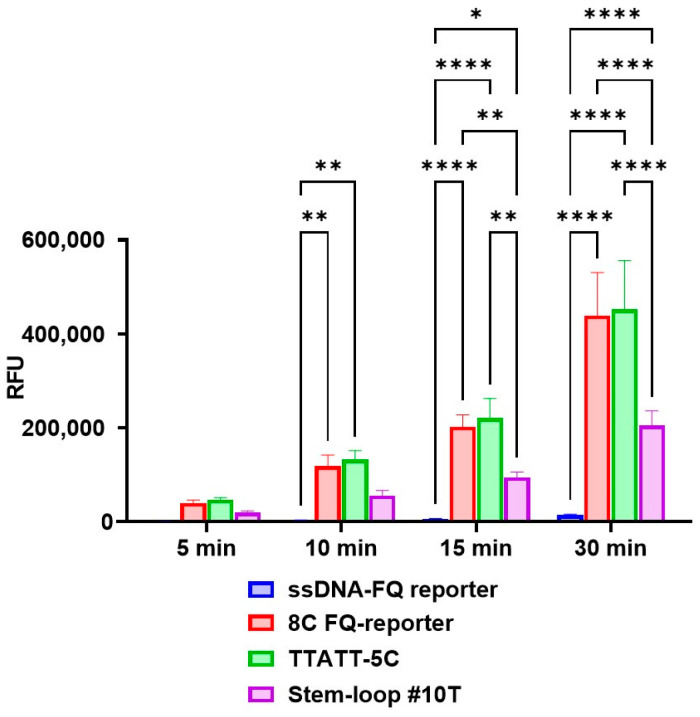
Fluorescence signal generated in the developed CRISPR/Cas12a system for detection of the target gene using the guide RNA crRNA_*bla*_OXA-1__63. A 1.25 × 10^0^ copy number of the *bla*_OXA-1_-encoding model matrix was added per pre-amplification step in all conditions. Error bars represent the standard error of the mean (n = 3). RFU = relative fluorescence units. Asterisks denote statistical significance (* *p* ≤ 0.05, ** *p* ≤ 0.01, **** *p* ≤ 0.0001).

Our comparative analysis revealed significant performance differences among the reporter systems. As early as the 10 min time point, reactions incorporating the TTATT-5C and 8C FQ-reporters demonstrated substantially stronger fluorescent signals compared with the conventional ssDNA-FQ system (*p* ≤ 0.01). This enhancement became more pronounced by the 15 min interval, with both optimized reporters showing superior performance relative to both the ssDNA-FQ (*p* ≤ 0.0001) and Stem-loop #10T (*p* ≤ 0.01) systems. Notably, the Stem-loop #10T reporter itself exhibited significantly greater signal intensity than the ssDNA-FQ control at both the 15 min (*p* ≤ 0.05) and 30 min (*p* ≤ 0.0001) time points (Figure 3). Complete quantitative comparisons of reporter performance characteristics are presented in Table S6 and Figure 3.

**Figure 4:**
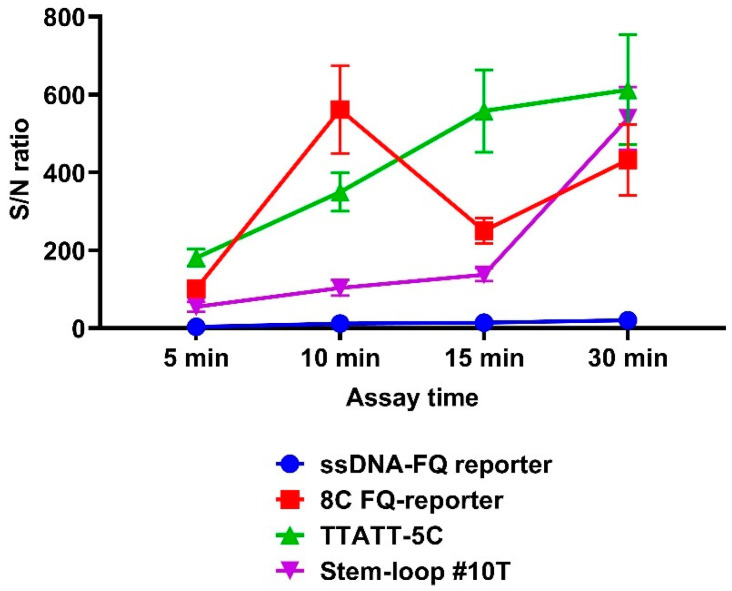
Signal-to-noise ratio of the developed CRISPR/Cas12a system for detection of the target gene using the guide RNA crRNA_*bla*_OXA-1__63. A 1.25 × 10^0^ copy number of the *bla*_OXA-1_-encoding model matrix was added per pre-amplification step in all conditions. Error bars represent the standard error of the mean (n = 3).

Notably, all improved fluorescent reporter molecules achieved a mean signal-to-noise (S/N) ratio exceeding 50 within the first 5 min of the assay, with values surpassing 400 by the 30 min time point (Figure 4). Comparative analysis revealed that, at the 5 min interval, only the TTATT-5C reporter demonstrated significantly higher S/N ratios compared with the conventional ssDNA-FQ reporter (*p* = 0.0082). This performance differential became more pronounced by the 10 min mark, where both the 8C FQ-reporter and TTATT-5C systems showed substantially elevated S/N ratios relative to both the ssDNA-FQ (*p* < 0.0001) and Stem-loop #10T reporters (*p* < 0.0001 and *p* = 0.0002, respectively). Complete statistical comparisons of reporter molecule performance are provided in Table S7.

In 2022, Lee et al. reported the development of TTATT-5C, a high-efficiency DNA reporter demonstrating a 100-fold stronger fluorescence signal compared with conventional ssDNA-FQ reporters [17]. When implemented in our CRISPR/Cas12a system for detection of single-copy *bla*_OXA-1_ targets, TTATT-5C yielded a 45-fold enhancement in fluorescence signal and a 30-fold improvement in S/N ratio relative to ssDNA-FQ reporters during the initial 5 min assay window.

Lv et al. subsequently developed the 8C FQ-reporter, though quantitative comparisons with conventional systems were not provided in the original publication [16]. Our experimental implementation revealed that this reporter generated a 35-fold stronger fluorescence signal and a 15-fold greater S/N ratio than ssDNA-FQ controls during 5 min assays.

Among hairpin DNA reporters, Stem-loop #10T (developed by Rossetti et al. [18]) demonstrated superior performance in our system, producing a 13-fold greater fluorescence intensity and 10-fold higher S/N ratios than ssDNA-FQ reporters within the 5 min timeframe.

While all enhanced reporters outperformed conventional ssDNA-FQ systems in standard Cas12 reaction buffers, we further investigated the impact of buffer composition on assay performance. We compared conventional 10× reaction buffer [23,24,25] and modified 10× HOLMES Buffer 1 [16] (See Section 2.1 for complete formulations).

**Figure 5:**
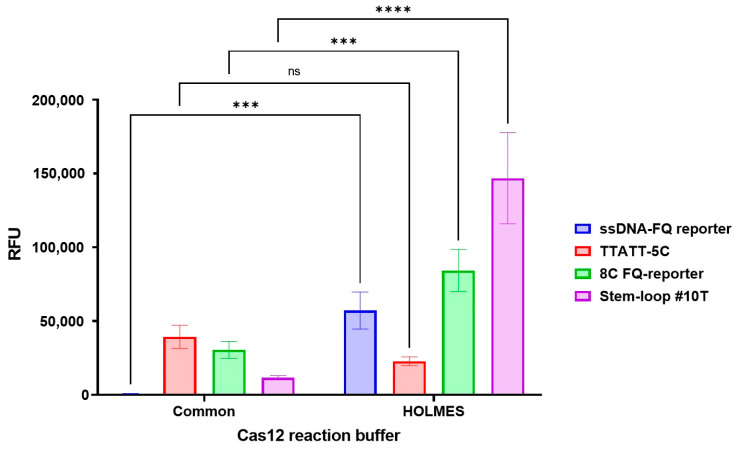
Fluorescence signal generated in the developed CRISPR/Cas12a system for detection of the target gene using the guide RNA crRNA_*bla*_OXA-1__63. A 1.25 × 10^0^ copy number of the *bla*_OXA-1_-encoding model matrix was added per pre-amplification step in all conditions. The assay duration was 5 min. Error bars represent the standard error of the mean (n = 3). RFU = relative fluorescence units. Asterisks denote statistical significance (*** *p* ≤ 0.001, **** *p* ≤ 0.0001, ns *p* > 0.05).

Implementation of HOLMES Buffer 1 significantly enhanced fluorescence signals across all reporter systems at the 5 min time point (ssDNA-FQ: *p* = 0.0004; 8C FQ-reporter: *p* = 0.0006; Stem-loop #10T: *p* < 0.0001). Optimal performance was achieved using HOLMES Buffer 1 with Stem-loop #10T reporter (Figure 5). Complete statistical analyses are presented in Table S8.

**Figure 6:**
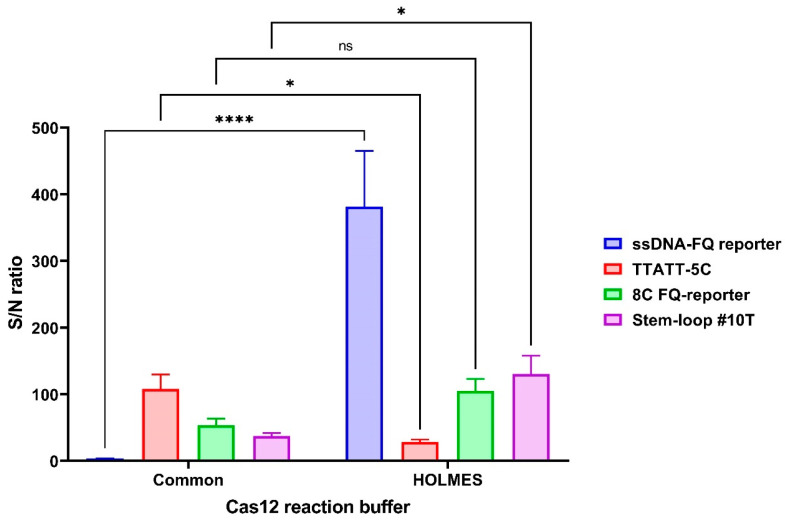
Signal-to-noise ratio of the developed CRISPR/Cas12a system for detection of the target gene using the guide RNA crRNA_*bla*_OXA-1__63. A 1.25 × 10^0^ copy number of the *bla*_OXA-1_-encoding model matrix was added per pre-amplification step in all conditions. The assay duration was 5 min. Error bars represent the standard error of the mean (n = 3). Asterisks denote statistical significance (* *p* ≤ 0.05, **** *p* ≤ 0.0001, ns *p* > 0.05).

Our analysis revealed that only the ssDNA-FQ reporter in standard Cas12 reaction buffer failed to meet the detection threshold (S/N ratio ≤ 5) at the 5 min time point. Buffer optimization significantly impacted reporter performance: while the TTATT-5C reporter showed a moderate 3.78-fold reduction in the S/N ratio when using HOLMES Buffer 1 (*p* = 0.0370), the Stem-loop #10T reporter demonstrated a 3.48-fold enhancement (*p* = 0.0129). Most notably, implementation of HOLMES Buffer 1 produced a dramatic >100-fold improvement in the ssDNA-FQ reporter’s S/N ratio (Figure 6). The optimal combination proved to be HOLMES Buffer 1 with ssDNA-FQ reporter, yielding the highest overall S/N ratio (Figure 6). Complete statistical comparisons of S/N ratios across experimental conditions are provided in Table S9.

### 3.4. Detecting Bla_OXA-1_ Gene in Real Samples

To assess the diagnostic performance of our optimized assay, we analyzed 50 *bla*_OXA-1_-positive genomic DNA samples isolated from clinically relevant strains (*E. cloacae*, *E. coli*, *K. pneumoniae*, *M. morganii*, *P. mirabilis*, *S. enterica*, and *S. marcescens*), along with 11 *bla*_OXA-1_-negative control samples (*A. baumannii*, *E. faecalis*, *E. faecium*, *P. aeruginosa*, *S. enterica*, and *S. aureus*). All assays were performed using HOLMES Buffer 1 and the three most effective fluorescent reporters identified during optimization: the ssDNA-FQ reporter, 8C FQ-reporter, and Stem-loop #10T.

**Figure 7:**
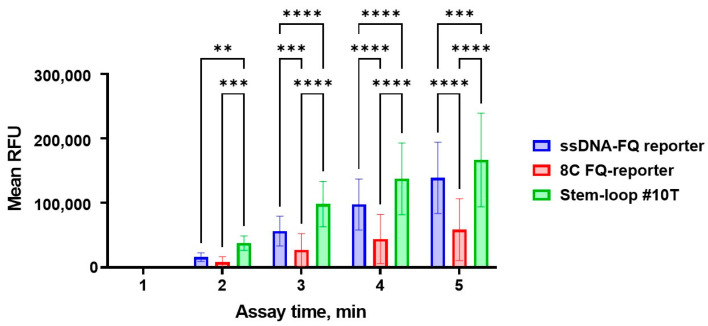
Fluorescence signal generated in the developed CRISPR/Cas12a system for detection of the target gene using the guide RNA crRNA_*bla*_OXA-1__63. A 1 µL volume of the *bla*_OXA-1_-positive genomic DNA sample was added per pre-amplification step in all conditions. The assay duration was 5 min. Error bars represent the standard error of the mean (n = 50). RFU = relative fluorescence units. Asterisks denote statistical significance (** *p* ≤ 0.01, *** *p* ≤ 0.001, **** *p* ≤ 0.0001).

**Figure 8:**
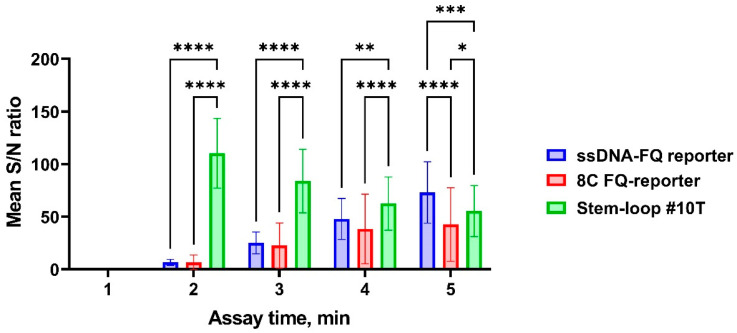
Signal-to-noise ratio of the developed CRISPR/Cas12a system for detection of the target gene using the guide RNA crRNA_*bla*_OXA-1__63. A 1 µL volume of the *bla*_OXA-1_-positive genomic DNA sample was added per pre-amplification step in all conditions. The assay duration was 5 min. Error bars represent the standard error of the mean (n = 50). Asterisks denote statistical significance (* *p* ≤ 0.05, ** *p* ≤ 0.01, *** *p* ≤ 0.001, **** *p* ≤ 0.0001).

All reporter systems successfully detected *bla*_OXA-1_-positive samples. Quantitative analysis revealed that the ssDNA-FQ and Stem-loop #10T reporters generated the highest fluorescence signals (Figure 7), while the ssDNA-FQ reporter demonstrated optimal signal-to-noise characteristics (Figure 8). Complete statistical comparisons of fluorescence signals and S/N ratios across reporter systems are presented in Figure 7 and Figure 8.

**Figure 9:**
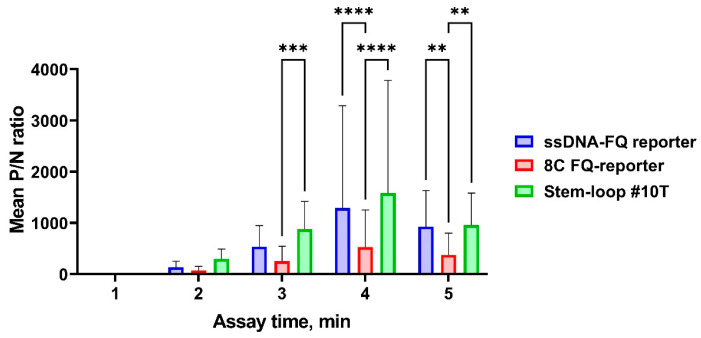
Positive-to-negative signal ratio of the developed CRISPR/Cas12a system for detection of the target gene using the guide RNA crRNA_*bla*_OXA-1__63. A 1 µL volume of both *bla*_OXA-1_-positive and *bla*_OXA-1_-negative genomic DNA samples was added per pre-amplification step in all conditions. The assay duration was 5 min. Error bars represent the standard error of the mean (n = 50 for *bla*_OXA-1_-positive samples; n = 11 for *bla*_OXA-1_-negative samples). Asterisks denote statistical significance (** *p* ≤ 0.01, *** *p* ≤ 0.001, **** *p* ≤ 0.0001).

Furthermore, we assessed the positive-to-negative (P/N) signal ratio for both *bla*_OXA-1_-positive samples and 11 *bla*_OXA-1_-negative genomic DNA controls. Our analysis revealed optimal discrimination capability when employing either the ssDNA-FQ reporter or Stem-loop #10T reporter systems, as evidenced by their superior P/N ratios (Figure 9). Complete statistical comparisons of mean P/N signal ratios across all reporter systems are presented in Figure 9.

## 4. Discussion

The *bla*_OXA-1_ gene is prevalent among ESBL-producing microorganisms spanning multiple bacterial families, including *Enterobacteriaceae*, *Morganellaceae*, *Pasteurellaceae*, *Moraxellaceae*, *Aeromonadaceae*, *Pseudomonadaceae*, *Yersiniaceae*, and *Vibrionaceae*. Current methodologies for ESBL detection, particularly those targeting OXA-1-producing microorganisms, remain constrained by their time-intensive nature, dependence on specialized equipment, and requirement for highly trained personnel.

CRISPR/Cas systems present significant opportunities for developing diverse point-of-care diagnostic tools (with over 20 distinct diagnostic platforms utilizing CRISPR/Cas9, CRISPR/Cas12, or Cas13 reported in the literature to date [12,26]), though these technologies currently remain primarily investigational rather than clinically implemented. CRISPR/Cas-based pathogen detection platforms exhibit notable advantages, including operational simplicity, exceptional specificity, high sensitivity (often reaching ultrasensitive detection thresholds), and the capacity to identify clinically relevant concentrations of pathogen-specific nucleic acids.

The standard CRISPR/Cas detection paradigm employs a two-step process involving initial pre-amplification of target nucleic acid sequences through various methodologies such as the PCR, LAMP, helicase-dependent amplification, RPA, strand displacement amplification, nucleic acid sequence-based amplification, transcription-mediated amplification, nicking enzyme-mediated amplification, or rolling circle amplification, followed by CRISPR/Cas-mediated detection of amplification products [23,24,25]. Among these, RPA has emerged as the most frequently employed pre-amplification method when coupled with CRISPR/Cas detection systems [26], with subsequent detection utilizing various CRISPR effectors including Cas9, Cas12a/b, Cas13, and Cas14 [12,26]. The mentioned-above CRISPR/Cas-based nucleic acid detection platforms achieve specificity through a dual-validation approach: (1) initial target selection via amplification with gene-specific oligonucleotides, followed by (2) computational validation of guide RNA specificity during assay design (see Table S2 for representative examples; specificity score is from 0–100 and higher is better).

In this study, we present a novel CRISPR/Cas12a-based detection system coupled with PCR for sensitive identification of OXA-1-bearing microorganisms. The assay comprises two principal components: a pre-amplification step utilizing conventional PCR methodology, followed by a nucleic acid detection step that exploits the nonspecific single-stranded DNA hydrolysis capability of CRISPR/Cas12a acting upon fluorescent reporter molecules.

Our PCR-coupled CRISPR/Cas12a fluorescence detection system demonstrates exceptional sensitivity, reliably detecting as few as 1.25 copies of the *bla*_OXA-1_ gene per reaction, representing a significant advancement over previously reported methods. While the existing literature documents the detection of *bla*_OXA-1_ in biological specimens [27,28], it fails to report method sensitivity thresholds [27,28]. Notably, Probst et al. (2021) described a PCR-based detection system for *bla*_OXA_-related genes with a sensitivity of 3.2 copies per reaction [29], which our method substantially surpasses.

The complete assay requires approximately 70 min, with 65 min allocated for pre-amplification and a remarkably efficient 5 min detection phase. This temporal optimization was achieved through systematic refinement of multiple detection parameters, including guide RNA selection [22,23,24,25] and optimization of both fluorescent reporter molecules [13,16,17,18] and reaction buffer composition.

The fluorescent reporter molecule constitutes a critical determinant of assay performance in CRISPR/Cas12a systems. Recent investigations have identified several DNA reporters that significantly outperform conventional ssDNA-FQ reporters [16,17,18]. Implementation of these enhanced reporter molecules has yielded multiple benefits, including reduced assay duration, improved method resolution, and substantially increased fluorescence values and signal-to-noise ratios.

HOLMES Buffer 1 incorporates several key modifications compared with standard Cas12 reaction buffers, including the addition of spermidine, PEG, DTT, Triton^®^ X-100, and glycine. Spermidine, a polyamine nucleic acid-binding agent, functions to neutralize DNA charge and has been shown to enhance interaction between distal DNA segments in various enzymatic systems [30,31]. For instance, spermidine improves the fidelity of *Escherichia coli* CRISPR Cas1-Cas2 integrase activity [32]. Polyethylene glycol (PEG) serves as a molecular crowding agent that stabilizes macromolecules against thermal denaturation while accelerating protein folding and nucleic acid renaturation, ultimately increasing enzymatic reaction rates and potentially modifying reaction products [33,34,35,36,37,38]. The reducing agent DTT maintains protein activity and is commonly incorporated in molecular biology enzyme buffers, including those for CRISPR/Cas protein storage [39]. Triton X-100, a nonionic surfactant known to enhance Cas12 system sensitivity through mechanisms yet to be fully elucidated [16,40], and glycine, an amino acid osmolyte crucial for protein stabilization [41], complete the optimized buffer formulation.

The synergistic action of these components dramatically improves CRISPR/Cas12a fluorescence assay performance, reducing processing time while enhancing resolution, consistent with previous findings [16]. Remarkably, our optimized system achieves signal-to-noise ratios of 400–600, representing a substantial improvement over previously reported values (S/N ≤ 15) for comparable assays [22].

The assay successfully distinguished blaOXA-1-positive clinical isolates (*E. cloacae*, *E. coli*, *K. pneumoniae*, *M. morganii*, *P. mirabilis*, *S. enterica*, and *S. marcescens*) from negative controls (*A. baumannii*, *E. faecalis*, *E. faecium*, *P. aeruginosa*, *S. enterica*, and *S. aureus*), achieving an exceptional positive-to-negative signal ratio of 750.

Three key parameters demand careful optimization when developing CRISPR/Cas12a systems for single-copy nucleic acid detection: (i) guide RNA selection, (ii) reporter molecule choice, and (iii) reaction buffer composition. Attention to these factors enables achievement of optimal performance metrics, including high fluorescence signals and superior signal-to-noise ratios.

We have developed a highly sensitive and rapid CRISPR/Cas12a-based detection system for the *bla*_OXA-1_ gene. Our assay demonstrates significant improvements in detection speed, sensitivity, and operational simplicity compared with conventional methods. However, further optimizations remain possible.

The primary enhancement would involve replacing PCR with recombinase polymerase amplification (RPA) or other isothermal amplification techniques during the pre-amplification step. Based on our experimental data [42], implementing RPA could reduce total analysis time by nearly half. Integration of RPA with our CRISPR/Cas12a detection system would decrease the assay duration from 70 min to approximately 40 min—notably faster than standard CRISPR/Cas-based analyses requiring 50–300 min [26]. Furthermore, RPA implementation would improve field-deployability by eliminating dependency on specialized thermal cycling equipment, making the assay more accessible in resource-limited settings. Looking ahead, we aim to develop a single-tube system integrating both pre-amplification and detection steps. Such closed-tube implementation would minimize risks of carryover contamination during amplification while further streamlining the workflow.

These planned optimizations—RPA integration and single-tube implementation—will streamline the assay into a sub-40 min, field-adaptable system with minimized contamination risks, positioning it as a next-generation tool for global AMR surveillance.

## 5. Conclusions

This study presents the development of a PCR-coupled CRISPR/Cas12a fluorescence assay designed for the detection of *bla*_OXA-1_-positive microorganisms with exceptional sensitivity and specificity. The assay architecture consists of two sequential phases: an initial pre-amplification step followed by a detection phase. The detection mechanism exploits the trans-cleavage activity of CRISPR/Cas12a, which mediates nonspecific hydrolysis of single-stranded DNA molecules, including fluorescent reporter molecules. Through systematic optimization of three critical components—crRNA design, fluorescent reporter molecule selection, and detection buffer composition—we have achieved outstanding analytical performance.

The optimized assay demonstrates remarkable sensitivity, capable of detecting as few as 1.25 copies of the *bla*_OXA-1_ gene per reaction within a total processing time of 70 min. This duration comprises 65 min for pre-amplification and an exceptionally rapid 5 min detection phase. The assay’s efficacy has been validated through successful detection of both model matrix samples containing precisely 1.25 copies of the *bla*_OXA-1_ gene and genomic DNA isolated from clinically relevant *bla*_OXA-1_-positive microorganisms.

The technological foundation of this assay holds significant promise for the development of novel point-of-care diagnostic kits that combine high sensitivity and specificity with operational simplicity. Unlike conventional methods, these potential diagnostic applications would not require specialized high-tech equipment or highly trained personnel. Further refinements could substantially enhance the assay’s practicality and field-deployability. Potential improvements include integration of isothermal amplification methodologies to eliminate dependence on traditional PCR instrumentation and implementation of single-tube systems to prevent carryover contamination during amplification. Such advancements would facilitate the assay’s adoption by surveillance and monitoring services as a contemporary molecular epidemiological tool.

## Figures and Tables

**Table 1:** The polymerase chain reaction (PCR) primers for *bla*_OXA-1_ pre-amplification, the guide RNAs (crRNAs), and the single-stranded DNA (ssDNA) reporter molecules for the CRISPR/Cas12a fluorescence assay.

Name	Function	Oligonucleotide Sequence	Reference
crRNA_*bla*_OXA-1__63	LbCpf1 crRNA	5′ AAUUUCUACUAAGUGUAGAUGGUUAUUUCUUGCGAAACCC 3′	This study
crRNA_*bla*_OXA-1__185	LbCpf1 crRNA	5′ AAUUUCUACUAAGUGUAGAUAAGCUACUUUCGAGCCAUGC 3′	This study
crRNA_*bla*_OXA-1__221	LbCpf1 crRNA	5′ AAUUUCUACUAAGUGUAGAUCGCAGGAAUUGAAUUUGUUC 3′	This study
OXA-1_for_45	PCR primer	5′ GGAATGGAGATCTGGAACAGCAATCATACACC 3′	This study
OXA-1_rev_48	PCR primer	5′ ATCCAGATCTTGTAGATACATGTTCTCTATGG 3′	This study
OXA-1_for_2	PCR primer	5′ AGCAATCATACACCAAAGACG 3′	This study
OXA-1_rev_1	PCR primer	5′ TGGCTGAGTTTTTAACTGGG 3′	This study
ssDNA-FQ reporter	ssDNA reporter	5′ FAM-TTATT-BHQ1 3′	[13]
8C FQ-reporter	ssDNA reporter	5′ FAM-CCCCCCCC-BHQ1 3′	[16]
TTATT-5C	ssDNA reporter	5′ FAM-TTATTCCCCC-BHQ1 3′	[17]
Stem-loop #10T	ssDNA reporter	5′ FAM-CTCTCATTTTTTTTTTAGAGAG-BHQ1 3′	[18]

## Data Availability

The original contributions presented in this study are included in this article; further inquiries can be directed to the corresponding author.

## Supplementary Materials

The following supporting information can be downloaded at https://www.mdpi.com/article/10.3390/cimb47040238/s1.
